# Virtual Reality–Guided Reaching During Treadmill Walking Alters Dynamic Stability in People with Stroke

**DOI:** 10.21203/rs.3.rs-10361445/v1

**Published:** 2026-07-25

**Authors:** Hanieh Pazhooman, Ricardo Vega, Bob Dirmish, Brian D. Schmit

**Affiliations:** Medical College of Wisconsin; Medical College of Wisconsin; Kobuk Technologies LLC; Marquette University

**Keywords:** Reaching, Balance, Stroke Rehabilitation, Virtual Reality, Whole-body Angular Momentum

## Abstract

**Background:**

This study was designed to determine how goal-directed reaching during virtual reality (VR)-based treadmill walking affects dynamic balance in stroke survivors. Frontal-plane whole-body angular momentum (H) was used to quantify dynamic balance during gait.

**Methods:**

Twenty individuals with stroke were enrolled in this study, and 16 were included in the analysis. Participants completed three blocks of a VR-based reaching task while walking on a treadmill after familiarization with both the VR system and treadmill walking. Kinematic data were collected using a markerless motion capture system (Theia3D, Theia Markerless Inc., Kingston, ON, Canada). Stride-level frontal-plane range of angular momentum (H_R_) was computed, and segmental contributions to H were analyzed.

**Results:**

Frontal-plane H_R_ significantly increased during the preparatory, reaching, and post-reaching strides compared with non-reaching strides at three walking speeds. H_R_ was also greater for contralateral than ipsilateral targets. Segmental analysis revealed that the trunk and reaching arm contributed most strongly to changes in H. Reaching was also accompanied by changes in stride time, length, and width.

**Conclusion:**

Reaching during walking imposes greater frontal-plane balance demands on stroke survivors, primarily through trunk and reaching-arm contributions. These findings support further investigation of task-specific, internally generated challenges as a component of VR-based gait rehabilitation.

## Introduction

Balance impairments during walking remain a major clinical concern after stroke, with up to 83% of survivors experiencing persistent mobility limitations (1). Fear of falling further restricts activity and quality of life (2), and a substantial proportion of falls occur during walking and other mobility tasks (3). Importantly, walking in daily life rarely occurs in isolation: individuals must identify targets, allocate attention, and perform goal-directed actions while maintaining stability. For dual task movements, locomotion and prehension are coordinated, with adaptations in stepping during object acquisition (4, 5). Such coupling suggests that upper-limb actions during walking impose additional mechanical and attentional demands, which is important for walking stability after stroke. However, the effects of goal-directed reaching on dynamic balance while walking in stroke survivors remain poorly characterized. Thus, the purpose of this study was to examine how reaching during walking influences dynamic balance control after stroke.

Conventional rehabilitation can improve balance, yet recovery of gait stability is often incomplete. Persistent instability is common even among individuals who regain independent ambulation (6), suggesting that standard therapies may not adequately challenge stability during walking. Virtual reality (VR)- and augmented reality (AR)-based gait training have emerged to address this limitation by manipulating visual feedback and task demands (7–9). Systems such as the C-Mill incorporate visually guided stepping and obstacle negotiation to help recover walking function and have demonstrated improvements in clinical mobility outcomes (10, 11). Further, treadmill training augmented with immersive or context-rich environments can enhance dynamic balance and gait outcomes compared with treadmill training alone (12). Nevertheless, most VR/AR paradigms emphasize external tasks, such as obstacle avoidance or visually guided foot placement. As a result, key aspects of dynamic balance regulation under realistic walking conditions may be insufficiently trained.

Reaching is a common functional activity during walking, with a biomechanical influence on gait stability. In healthy adults, reaching is superimposed onto rhythmic gait through parallel or hierarchically organized control processes that preserve overall stability (4, 5, 13). However, adjustments in step timing and arm–trunk coordination indicate that reaching tasks alter locomotor mechanics critical to balance (14). In stroke survivors, arm movement during treadmill training is feasible and can improve clinical outcomes (15), yet underlying biomechanical effects remain somewhat unexplored. Mechanically, reaching redistributes upper-body mass and produces segmental motion, introducing an internal perturbation requiring compensation to maintain whole-body stability.

To quantify the effects of such internal perturbations, a biomechanical measure of whole-body stability is needed. Whole-body angular momentum (H) about the center of mass reflects the net rotational state arising from segmental motion and ground reaction forces (16). During steady walking, H is regulated through intersegmental strategies that limit excessive whole-body rotation (16, 17). Frontal-plane H is closely linked to mediolateral balance control via foot placement and center-of-pressure. In stroke survivors, impaired regulation of frontal-plane H has been associated with poorer clinical balance and altered control of lateral foot placement (18, 19). Moreover, H is sensitive to increased task demands and balance perturbations, supporting its utility as a measure of dynamic balance regulation beyond steady-state gait (20, 21). Because reaching involves substantial arm and trunk motion, examining frontal-plane H during reaching provides a framework for evaluating how internal perturbations alter balance in people with stroke.

Building on this framework, we investigated whether goal-directed reaching during walking disrupts dynamic balance in stroke survivors. Using a VR-based treadmill paradigm to elicit reaching, we quantified stride-level changes in frontal-plane H during reaching and non-reaching strides and examined segmental contributions to H. We hypothesized that reaching would increase frontal-plane H and alter segmental contributions, particularly from the upper extremities and trunk, reflecting redistribution of angular momentum during internal perturbations.

## Methods

### Participants

Twenty participants (10 females, 10 males) were recruited through a HIPAA-compliant, secure REDCap database managed by the Stroke Rehabilitation Center of Southeast Wisconsin. Inclusion criteria were: age ≥ 18 years, ability to provide informed consent, ≥ 6 months post-diagnosis of unilateral middle cerebral artery subcortical stroke, and a Functional Gait Assessment (FGA) score < 27. Exclusion criteria included peripheral vascular disease, chronic back pain, history of substance abuse or head trauma, comorbid neurological disorders, inability to follow three-step commands, visual-spatial neglect, or other significant orthopedic, neurological, cardiopulmonary, or metabolic conditions limiting ambulation. All participants provided written informed consent before study participation.

### Procedures

**Figure 1:**
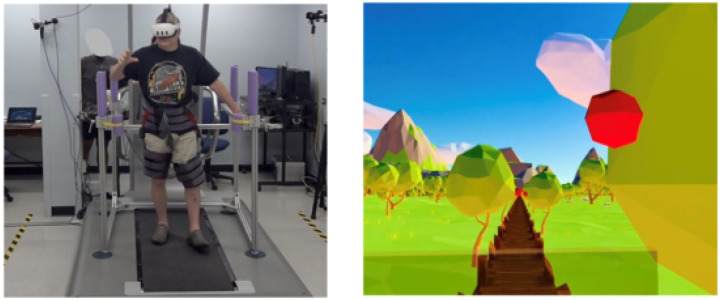
The apparatus for assessing virtual reality–guided reaching during treadmill walking. Right: Virtual scene from KBT’s VR training program. The treadmill surface is portrayed as a walkway, with apple trees growing along the walkway. An avatar of the hand is displayed for the participant to guide towards the apples on the trees. Left: Participant set-up with harness and VR headset. The handrail was available for participants that typically used assistive devices for overground ambulation.

Participants completed clinical assessments followed by treadmill walking trials under baseline and virtual reaching conditions. Clinical measures included the Fugl-Meyer Assessment (upper and lower extremity), Berg Balance Scale (BBS), and Functional Gait Assessment (FGA) (22, 23). A safety harness was secured while participants walked on a treadmill (Balance Tutor^™^ BT100, MediTouch, Israel). Comfortable walking speed (CWS) was determined by gradually increasing treadmill speed until participants reported a comfortable pace. Participants were then oriented to the VR treadmill system (Fig. 1). Two baseline trials were performed at CWS: one without VR and one using a Meta Oculus Quest 2 headset with Kobuk Technologies software. Participants then completed three 9-minute VR walking blocks. In each block, 45 randomized virtual targets (apples) appeared on trees. Participants reached with their nonparetic hand to collect targets and place them into a basket positioned anteriorly. Targets were categorized by height and by position (ipsilateral or contralateral relative to the nonparetic hand). Walking speed was randomized across blocks at 60%, 80%, and 100% CWS. Rest breaks were provided as needed. Markerless motion capture (Theia3D, Theia Markerless Inc., Kingston, ON, Canada) recorded full-body kinematics at 60Hz.

### Data processing

Kinematic data were processed to quantify whole-body and segmental angular momentum responses to reaching. A 16-segment three-dimensional model was constructed in Visual3D. Whole-body angular momentum (H) about the center of mass in the frontal plane was calculated as:

$$\text{H}=\sum_{\text{i}=1}^{\text{n}}\left[\left({\text{r}}_{\text{i}}-{\text{r}}_{\text{com}}\right)\times {\text{m}}_{\text{i}}\left({\text{V}}_{\text{i}}-{\text{V}}_{\text{com}}\right)+{\text{I}}_{\text{i}}\omega_{\text{i}}\right]$$

**Figure 2:**
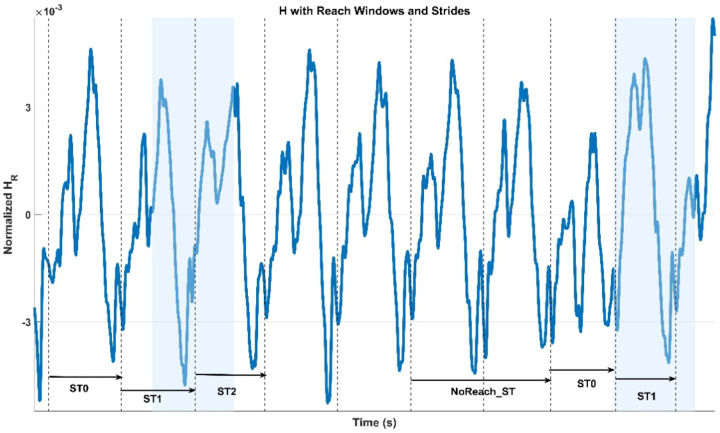
Stride and reach windows. The pale blue boxes indicate the reach windows. Vertical dashed lines show the heel strikes of each stride. Time is reported in seconds. NoReach_ST are strides with no reach prior to ST0, ST0 is the stride prior to reach, ST1 is the first stride of the reach and ST2 is the second stride of the reach.

Where the r_i_ and V_i_ are the position and velocity of the i-th segment’s center of mass (CoM) and r_com_ and V_com_ are the position and velocity of the whole-body’s CoM. *ω*_i_, m_i_, and I_i_ are the angular velocity, and mass and moment of inertia of the i-th segment, respectively, and n is the number of segments. Data were low-pass filtered using a fourth-order Butterworth filter (cutoffs: 6 Hz for hand position and H; 5 Hz for heel velocity). H was normalized to body mass (kg), height (m), and $\sqrt{\text{g}\times \text{l}}$, where g is the gravitational acceleration, and l is participant’s height (24). Nonparetic strides were identified using heel velocity (15). Reach-events were defined based on peak reaching hand position in mediolateral, anteroposterior, and vertical directions. To investigate the participants’ response to reach, the range of H (H_R_) was calculated as the difference of the maximum and minimum of H within the stride prior to each reach-event (ST0), the first stride containing each reach-event (ST1), the stride after each reach-event (ST2), and strides between the second stride after each reach event to the following ST0 (NoReach_ST) (Fig. 2).

To examine segmental contributions to H, the angular momentum (AM_seg_) of the reaching arm (RA), non-reaching arm (nRA), reaching leg (RL), non-reaching leg (nRL), and trunk segments were computed in MATLAB (The MathWorks Inc., Natick, MA) as:

$${\text{AM}}_{\text{seg}}=\left[\left({\text{r}}_{\text{seg}}-{\text{r}}_{\text{com}}\right)\times {\text{m}}_{\text{seg}}\left({\text{V}}_{\text{seg}}-{\text{V}}_{\text{com}}\right)+{\text{I}}_{\text{seg}}\omega_{\text{seg}}\right]$$

Arm segments consisted of upper arm, forearm and hand components. Leg segments consisted of thigh, shank, and foot components. The trunk segment included pelvis and thorax components. Explained share (ES) was used to quantify how changes in segmental angular momentum aligned with changes in whole-body H at CWS:

$$\text{ES=}\frac{\int_{0}^{100}\left(\Delta \text{H}\left(\text{t}\right).{\text{AM}}_{\text{seg}}\left(\text{t}\right)\right)\Delta \text{t}}{\int_{0}^{100}\left(\Delta \text{H}\left(\text{t}\right).\Delta \text{H}\left(\text{t}\right)\right)\Delta \text{t}}$$

where *Δ* H(t) is the difference of H time-series for each test stride (ST0, ST1, and ST2) relative to the mean time series (NoReach_ST); and AM_seg_ (t) is the difference of each AM_seg_ time-series for each test stride (ST0, ST1, and ST2) from the mean NoReach_ST. ES quantifies how much of the change in the H is aligned with the change AM_seg_. Unlike traditional absolute contribution methods (25), ES accounts for both magnitude and directional coordination between segmental and whole-body angular momentum. Stride time, length, and width were also calculated for ST0, ST1, ST2, and NoReach_ST.

#### Statistical analysis

Statistical analyses were used to evaluate the effects of reaching stride, treadmill speed, and target position on measures of postural stability (H) and spatiotemporal measures of stepping. A three-way repeated-measures ANOVA tested the effects of stride (NoReach_ST, ST0, ST1, ST2), speed (60%, 80%, 100% CWS), and target position (ipsilateral vs contralateral) on H_R_. Post hoc analyses included two-way repeated-measures ANOVAs, simple main effects, and Bonferroni-adjusted paired t-tests as appropriate. Separate one-way repeated-measures ANOVAs examined ES differences across segments at each stride and target condition. One-way repeated-measures ANOVAs evaluated stride time, length, and width across stride conditions, with Bonferroni-adjusted paired t-tests for follow-up.

## Results

### Participant Characteristics

Four participants were excluded (one missing data, three handrail use). Sixteen participants (9 females, 7 males) were analyzed. Mean age was 58 ± 11 years and BMI 29.38 ± 5.54. Clinical scores were BBS: 50.65 ± 3.78, FGA: 19.65 ± 4.75, Fugl-Meyer LE: 27.35 ± 3.41, Fugl-Meyer UE: 48.6 ± 16.6. These scores indicate mild-to-moderate balance impairments consistent with study inclusion criteria.

### Effects of Reaching and Treadmill Speed on H_R_

We first examined whether reaching and treadmill speed influenced frontal-plane H_R_. There was no significant three-way interaction between reaching stride, treadmill speed, and target position on H_R_ (p = 0.23, η^2^ = 0.498). Among the two-way interactions for H_R_, only treadmill speed × stride was significant (p = 0.033, η^2^ = 0.172); the interactions of target position and treadmill speed (p = 0.413, η^2^ = 0.119) and target position and stride (p = 0.056, η^2^ = 0.184) were not significant.

**Figure 3:**
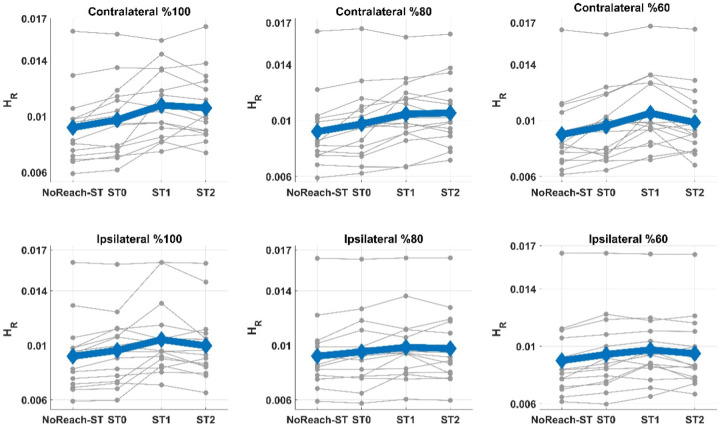
H_R_ in each stride. Blue lines show the group mean. The grey lines represent each participant’s mean H_R_. Contralateral and ipsilateral reach motions at three different gait speeds (100%, 80% and 60% CWS) are shown in separate panels.

Reaching stride demonstrated a significant simple main effect on H_R_ across all treadmill speeds. H_R_ significantly increased during ST0, ST1, and ST2 compared to NoReach_ST at 100% CWS (p = 0.003, η^2^ = 0.65), 80% of CWS (p = 0.005, η^2^ = 0.62), and 60% of CWS (p < 0.001, η^2^ = 0.74) (Fig. 3).

Pairwise comparisons showed that H_R_ was significantly higher in ST0, ST1, and ST2 compared to NoReach_ST at all speeds. H_R_ was also greater in ST1 than ST0 at 100% and 60% of CWS. No significant differences were found between ST2 and the other reaching strides at any speed (Table 1). Target position had a significant main effect on H_R_ (p = 0.037, η^2^ = 0.259). Post hoc paired t-tests indicated that H_R_ was significantly higher when reaching to contralateral targets compared to ipsilateral targets (p = 0.037, η^2^ = 0.57).

### Segmental Contributions (ES)

**Figure 4:**
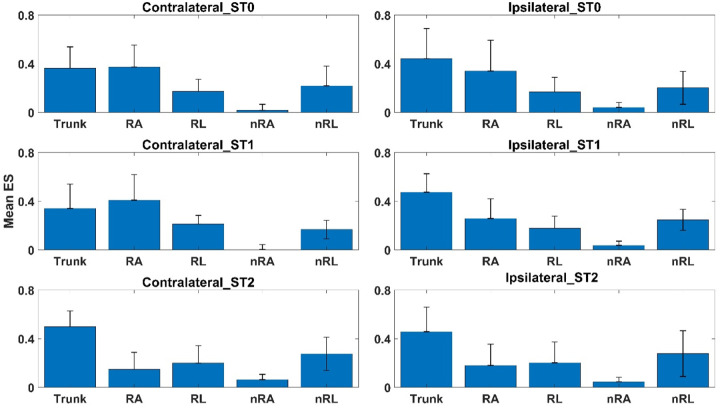
ES bar graph representation of body segments. Error bars are standard deviation across participants. RA=reaching arm, RL=leg on reaching side, nRA=non reaching arm, nRL=leg on non reaching side. Separate panels show ipsilateral and contralateral reach for three strides associated with the reach events (ST0, ST1 and ST2).

**Figure 5:**
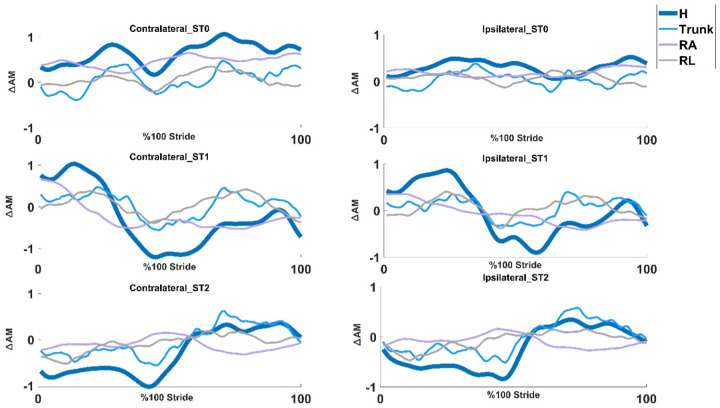
Time-normalized changes in frontal-plane H and the angular momentum (AM) of the trunk, reaching arm (RA), and reaching leg (RL) during ST0, ST1, and ST2 for contralateral and ipsilateral reaches. Dimensionless changes (ΔAM) are expressed relative to non-reaching strides, and each stride is normalized from 0% to 100%.

To understand the segmental angular momentum underlying H_R_ changes, ES was examined for each body segment. One-way repeated-measures ANOVAs revealed significant differences in ES among body segments at ST0, ST1, and ST2 for both contralateral (p < 0.001, η^2^ = 0.481–0.654) and ipsilateral targets (p < 0.001, η^2^ = 0.400–0.923) (Fig. 4). Detailed pairwise comparisons are presented in Table A1 (Appendix A). Across conditions, trunk and reaching arm (RA) segments contributed more strongly to H changes, whereas the non-reaching arm (nRA) showed consistently lower ES values. For contralateral targets: 1) RL ES was lower than RA and trunk at ST0, 2) RA and trunk exceeded nRL and RL at ST1 and 3) trunk exceeded nRL, RA, and RL at ST2. For ipsilateral targets: 1) trunk exceeded nRL and RL at ST0, 2) trunk exceeded nRL, RA, and RL at ST1 and 3) trunk remained greater than RA and RL at ST2. For reference, the mean time-normalized changes in H and the AM of the trunk, reaching arm, and reaching leg across ST0, ST1, and ST2 are shown for contralateral and ipsilateral reaches in Fig. 5.

### Spatiotemporal Adjustments

**Figure 6:**
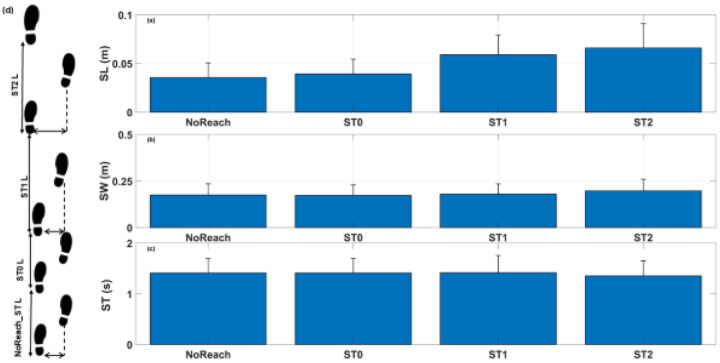
a: group means of stride length (SL). b: group means of step width (SW). c: group means of stride time (ST). d: spatiotemporal parameters illustrations. All error bars reflect standard deviation.

Finally, we examined spatiotemporal adaptations during reaching. One-way repeated measure ANOVAs showed stride time (p = 0.011, η^2^ = 0.236), stride length (p < 0.001, η^2^ = 0.815), and stride width (p < 0.001, η^2^ = 0.494) were significantly different between reaching strides (Fig. 6). Stride time was shortest during ST2. Stride length increased during ST1 and ST2 compared to NoReach_ST and ST0. Stride width was greater in ST2 compared to NoReach_ST and ST0 (Table 2). These findings suggest compensatory spatiotemporal adjustments during reaching.

## Discussion

The primary objective of this study was to investigate the effects of reaching on the control of dynamic stability during treadmill walking. Our findings demonstrated that reaching in a VR environment increased frontal-plane H across walking speeds at 100%, 80%, and 60% of participants’ CWS. The trunk and RA contributed the most to the observed changes in H, whereas nRA exhibited minimal contributions (Table A1). In addition, participants demonstrated adaptations in gait mechanics during reaching, characterized by shorter ST and longer SL compared to normal strides. Toward the end of the reaching movement, participants also adopted a wider stride, suggesting a compensatory strategy to enhance lateral stability.

### Reaching as an Internal Perturbation to Balance while Walking

Goal-directed reaching during walking acted as an internal perturbation to frontal plane balance in individuals with chronic stroke, as reflected by consistent increases in H_R_ in the frontal plane. H about the body’s COM is regulated during walking through coordinated cancellations among body segments to control rotational motion and maintain balance (17). H_R_ has also been shown to be associated with clinical balance measurements in stroke survivors, where greater frontal-plane H_R_ is correlated with lower scores on the BBS and the Dynamic Gait Index (DGI) (18). Reaching shifts the COM and alters H, acting as an internally induced perturbation that disrupts postural stability. Rapid arm movements and reaching tasks have been shown to induce anticipatory postural adjustments in lower limbs to compensate for these perturbations (26). Similarly, other functional tasks that create internal perturbations, such as sit-to-stand, shift the body’s COM and require postural adjustments to maintain balance (27). In our study, H_R_ was significantly greater in ST1 and ST2 compared with NoReach_ST, indicating that reaching altered H regulation and increased instability. We also observed that H_R_ increased in ST0 compared with NoReach_ST, suggesting that participants may begin to alter H and experience instability during the preparatory phase of reaching. Although no significant differences were observed between ST1 and ST2 or between ST2 and ST0, H_R_ tended to decrease from ST1 to ST2, which may reflect the initiation of balance recovery after the first stride of reach. At the same time, H_R_ remained elevated in ST2 compared with ST0, suggesting that the effects of reaching on stability may persist beyond the first stride of reach itself (Fig. 3). Together, these findings demonstrate that upper-limb actions superimposed on gait measurably disrupt dynamic balance after stroke.

### Segmental Distribution and Trunk-Mediated Control

Segmental analyses revealed that trunk angular momentum accounted for the largest proportion of stride-level changes in H during reaching, with the reaching arm contributing secondarily (Fig. 4). Reaching movements require additional postural adjustments to maintain dynamic balance during walking. Previous studies have shown that individuals adopt more conservative walking strategies and coordinate reaching with locomotion to better control stability (5, 14). In stroke survivors, trunk position sense is often impaired and anticipatory activation of bilateral trunk muscles is delayed (28, 29), which can lead to greater trunk displacement during reaching tasks (20). Temporal analysis of AM_seg_ revealed distinct coordination patterns across strides (Fig. 5). In ST0, changes in RL angular momentum preceded changes in H, suggesting the presence of anticipatory postural adjustments that likely prepare the system for the upcoming destabilization associated with reaching. In contrast, trunk angular momentum changes occurred concurrently with H, indicating that the trunk contributes primarily to the regulation and redistribution of angular momentum rather than initiating the movement. Notably, the RA exhibited an opposing pattern relative to the trunk and H, suggesting a counterbalancing role in mediolateral stability. During the reaching strides (ST1 and ST2), AM_seg_ changes across the trunk, RL, and H became more synchronized, highlighting increased intersegmental coupling during task execution, while the RA continued to exhibit an opposing contribution. These findings were consistent across both contralateral and ipsilateral reaching conditions, suggesting a task-dependent rather than limb-dependent control strategy. Additionally, the consistent timing of trunk angular momentum with H dynamics highlights the trunk’s role as a central contributor to mediolateral stability. Trunk adjustments may also interact with stepping mechanics, as step width and foot placement are known to adapt in response to changes in trunk motion to maintain balance (30). These findings reinforce the role of trunk control in mediolateral stability after stroke and highlight intersegmental coordination, rather than isolated limb motion, as a key determinant of dynamic balance during complex walking tasks.

### Implications for VR-Based Gait Training

Most VR-based gait paradigms emphasize externally imposed challenges such as obstacle negotiation, whereas the present findings demonstrate that internally generated reaching tasks systematically increase frontal-plane rotational demands. Previous VR-based gait studies have challenged stability in individuals post-stroke using tasks such as street crossing, park navigation, and virtual obstacle avoidance, or through unpredictable visual stimuli requiring rapid postural adjustments (7, 15, 31). These paradigms primarily introduce external perturbations that necessitate modifications in walking speed, foot placement, and stride length to maintain stability. In contrast, the current findings indicate that goal-directed reaching movements can serve as an internally generated perturbation. Specifically, contralateral targets elicited greater frontal-plane H_R_, reflecting increased rotational demands compared to ipsilateral targets. This suggests that target location can be used to systematically scale the magnitude of balance perturbations. Furthermore, the significant interaction between stride type and walking speed indicates that speed acts as an additional controllable parameter influencing task difficulty. Together, these findings highlight that VR-based gait training can be tailored by manipulating both target location and treadmill speed to systematically adjust perturbation magnitude. These results align with principles of task-specific training, which suggest that motor improvements are optimized when practice conditions closely match task demands. The current VR-based paradigm incorporates goal-directed reaching during treadmill walking, introducing functionally relevant demands that require coordination across body segments, anticipatory postural adjustments, and dynamic regulation of H. As such, incorporating goal-directed arm movements into VR treadmill training may better approximate the multitask demands of community ambulation, where individuals simultaneously walk and interact with their environment.

### Limitations and Future Directions

This study included individuals with mild-to-moderate impairments, which may limit the generalizability of the findings to those with more severe deficits. Additionally, participants performed reaching tasks only with the non-paretic arm; therefore, the effects of reaching with the paretic arm during walking remain unclear and warrant further investigation. The experimental protocol was conducted on a treadmill, and it is unknown whether these findings translate to overground walking conditions, where environmental and task demands differ. Moreover, the absence of a neurologically intact control group affects interpretation of the results. It is also important to consider that the trunk may not represent the dominant contributor to changes in whole-body angular momentum in the neurological intact individuals, as these individuals do not exhibit trunk movement deficits. Future studies should examine bilateral and paretic reaching, incorporate overground paradigms, and look at training paradigms to see if there are adaptations in stability control that translate to dynamic balance functional outcomes.

## Figures and Tables

**Table 1:** Stride H_R_ pairwise comparisons following the simple main effect tests

Speed	Comparisons	Mean ± SE (× 10^−3^)	P-value	d_z_
%100	NoReach_ST - ST0	−0.5 ± 0.13	0.014*	0.659
	NoReach_ST - ST1	−1.4 ± 0.27	< 0.001*	> 1
	NoReach_ST - ST2	−1.1 ± 0.25	0.004*	0.848
	ST0 - ST1	−0.92 ± 0.13	0.006*	0.799
	ST0 - ST2	−0.6 ± 0.23	0.188	0.344
	ST1 - ST2	0.32 ± 0.25	0.214	0.324
%80	NoReach_ST - ST0	−0.43 ± 0.14	0.045*	1.26
	NoReach_ST - ST1	−0.95 ± 0.2	0.002*	1.21
	NoReach_ST - ST2	−0.94 ± 0.21	0.002*	1.21
	ST0 - ST1	−0.52 ± 0.17	0.053	1.24
	ST0 - ST2	−0.51 ± 0.18	0.081	1.24
	ST1 - ST2	0.01 ± 0.014	1	0
%60	NoReach_ST - ST0	−0.52 ± 0.15	0.017*	0.67
	NoReach_ST - ST1	−1.5 ± 0.18	< 0.001*	> 1
	NoReach_ST - ST2	−6.8 ± 0.15	0.002*	0.933
	ST0 - ST1	−0. 62 ± 0.17	0.018*	0.663
	ST0 - ST2	−0.16 ± 0.18	1	0
	ST1 - ST2	0.46 ± 0.17	0.093	0.448

Effect sizes are reported as Cohen’s dz.

* indicates significant difference after Bonferroni adjustments. The p-values are Bonferroni corrected.

**Table 2:** Spatiotemporal metrics pairwise comparisons following the significant one-way repeated measure ANOVA

Parameters	Comparisons	Mean ± SE (× 10^−3^)	P-value	d_z_
Stride-Time	ST2-NoReach_ST	− 58 ± 19.6	0.005*	0.76
	ST2-ST0	− 56 ± 21.7	0.011*	0.67
	ST2-ST1	− 64 ± 21.6	0.005*	0.765
Stride-Length	ST1-NoReach_ST	24 ± 3.4	< 0.001*	1.7
	ST1-ST0	20 ± 3.0	< 0.001*	1.6
	ST2-NoReach_ST	30 ± 4.3	< 0.001*	1.74
	ST2-ST0	27 ± 4.9	< 0.001*	1.35
Stride-Width	ST2-NoReach_ST	22 ± 5.4	< 0.001*	1.07
	ST2-ST0	24 ± 6.2	< 0.001*	1.01

Effect sizes are reported as Cohen’s dz.

* indicates the significant difference after Bonferroni adjustments. The p-values aren’t Bonferroni corrected.

## Data Availability

The data generated and/or analyzed during this study are available from the corresponding author on reasonable requests.

## Appendix A

**Table A1:** Segments Contributions’ pairwise comparisons following the significant simple main effect

Strides	Comparisons	Mean ± SE	P-value	dz
**C_ST0**	nRA – nRL	−0.201 ± 0.043	0.003*	0.88
	nRA – RA	−0.355 ± 0.050	< 0.001*	> 1
	nRA – RL	−0.155 ± 0.028	< 0.001*	> 1
	nRA – trunk	−0.345 ± 0.042	< 0.001*	> 1
	nRL – RA	−0.155 ± 0.071	0.463	0.18
	nRL – RL	0.046 ± 0.058	1	0
	nRL – trunk	−0.144 ± 0.071	0.610	0.13
	RA – RL	0.201 ± 0.060	0.044*	0.55
	RA – trunk	0.011 ± 0.078	1	0
	RL – trunk	−0.190 ± 0.032	< 0.001*	> 1
**C_ST1**	nRA – nRL	−0.165 ± 0.017	< 0.001*	> 1
	nRA – RA	−0.407 ± 0.062	< 0.001*	> 1
	nRA – RL	−0.211 ± 0.021	< 0.001*	> 1
	nRA – trunk	−0.338 ± 0.044	< 0.001*	> 1
	nRL – RA	−0.241 ± 0.067	0.026*	0.62
	nRL – RL	−0.046 ± 0.029	1	0
	nRL – trunk	−0.173 ± 0.047	0.021*	0.64
	RA – RL	0.196 ± 0.056	0.034*	0.58
	RA – trunk	0.069 ± 0.097	1	0
	RL – trunk	−0.127 ± 0.056	0.374	0.23
**C_ST2**	nRA – nRL	−1.103 ± 0.037	< 0.001*	> 1
	nRA – RA	−0.574 ± 0.039	0.305	0.26
	nRA – RL	−0.797 ± 0.040	0.005*	0.82
	nRA – trunk	−2.185 ± 0.027	< 0.001*	> 1
	nRL – RA	0.529 ± 0.050	0.603	0.132
	nRL – RL	0.306 ± 0.062	1	0
	nRL – trunk	−1.082 ± 0.052	0.002*	0.93
	RA – RL	−0.223 ± 0.061	1	0
	RA – trunk	−1.611 ± 0.053	< 0.001*	> 1
	RL – trunk	−1.388 ± 0.052	< 0.001*	> 1
**I_ST0**	nRA – nRL	−0.161 ± 0.033	0.002*	0.93
	nRA – RA	−0.299 ± 0.068	0.005*	0.82
	nRA – RL	−0.128 ± 0.032	0.013*	0.7
	nRA – trunk	−0.402 ± 0.058	< 0.001*	> 1
	nRL – RA	−0.138 ± 0.085	1	0
	nRL – RL	0.033 ± 0.050	1	0
	nRL – trunk	−0.240 ± 0.070	0.036*	0.57
	RA – RL	0.172 ± 0.085	0.603	0.132
	RA – trunk	−0.102 ± 0.117	1	0
	RL – trunk	−0.274 ± 0.056	0.002*	0.93
**I_ST1**	nRA – nRL	−0.210 ± 0.022	< 0.001*	> 1
	nRA – RA	−0.221 ± 0.044	0.001*	1.01
	nRA – RL	−0.140 ± 0.030	0.003*	0.88
	nRA – trunk	−0.438 ± 0.032	< 0.001*	> 1
	nRL – RA	−0.011 ± 0.055	1	0
	nRL – RL	0.070 ± 0.036	0.680	0.1
	nRL – trunk	−0.227 ± 0.038	< 0.001*	> 1
	RA – RL	0.081 ± 0.057	1	0
	RA – trunk	−0.216 ± 0.065	0.046*	0.54
	RL – trunk	−0.298 ± 0.052	< 0.001*	> 1
**I_ST2**	nRA – nRL	−0.232 ± 0.050	0.003*	0.88
	nRA – RA	−0.134 ± 0.051	0.188	0.34
	nRA – RL	−0.156 ± 0.043	0.023*	0.63
	nRA – trunk	−0.412 ± 0.044	< 0.001*	> 1
	nRL – RA	0.098 ± 0.062	1	0
	nRL – RL	0.076 ± 0.085	1	0
	nRL – trunk	−0.180 ± 0.079	0.38	0.23
	RA – RL	−0.022 ± 0.070	1	0
	RA – trunk	−0.277 ± 0.086	0.055	0.52
	RL – trunk	−0.256 ± 0.060	0.007*	0.78

Effect sizes are reported as Cohen’s dz.

* indicates the significant difference after Bonferroni adjustments. The p-values are Bonferroni corrected. C_STX is stride X of the contralateral reach task, I_STX is stride X of the ipsilateral reach task. RA is reach-side arm segment, nRA is non-reach-side arm segment, RL is reach-side leg segment, nRL is non-reach-side leg segment.
